# Autoimmune markers for the diagnosis of rheumatoid arthritis in primary care: primary care diagnostic technology update

**DOI:** 10.3399/bjgp13X673919

**Published:** 2013-10

**Authors:** Kamal R Mahtani, Anne Miller, Oliver Rivero-Arias, Carl Heneghan, Christopher P Price, Matthew Thompson, Annette Plüddemann, Raashid Luqmani

**Affiliations:** Horizon Scanning Programme, Centre for Monitoring and Diagnosis, University of Oxford, Oxford.; Nuffield Orthopaedic Centre, Oxford University Hospitals NHS Trust, Oxford.; Health Economics Research Centre, Nuffield Department of Population Health, University of Oxford, Oxford and Red de Investigación de Servicios Sanitarios en Cronicidad (REDISSEC), Spain.; Horizon Scanning Programme, Centre for Monitoring and Diagnosis, University of Oxford, Oxford.; Horizon Scanning Programme, Centre for Monitoring and Diagnosis, University of Oxford, Oxford.; Horizon Scanning Programme, Centre for Monitoring and Diagnosis, University of Oxford, Oxford.; Horizon Scanning Programme, Centre for Monitoring and Diagnosis, University of Oxford, Oxford.; NIHR Biomedical Research Unit, University of Oxford, Oxford.

**Clinical Question**

Should GPs use anti-citrullinated peptide antibody testing instead of rheumatoid factor for diagnosing rheumatoid arthritis?

## BACKGROUND AND ADVANTAGES OVER EXISTING TECHNOLOGY

Early diagnosis and treatment of rheumatoid arthritis (RA) is important in preventing long-term damage and disability. RA should be suspected largely on the basis of clinical findings, such as persistent joint pain, swelling, and stiffness. Further investigations, particularly in primary care, may contribute to the diagnosis. Rheumatoid factor (RF) is an autoantibody associated with RA and its presence has traditionally been used to support the diagnosis. However, RF has a low specificity in primary care and cannot be used to rule in or rule out disease. In contrast, anti-citrullinated peptide antibody (ACPA) has emerged as an alternative serological test, as it has greater specificity and may be preferable to RF in the diagnosis of RA.^1^ However, it is not yet generally available in primary care.

## DETAILS OF TECHNOLOGY

RFs are autoantibodies directed against the Fc region of immunoglobulin IgG. RA is associated with the presence of RF in many, but not all cases. Raised levels are also found in other autoimmune diseases, for example, Sjogren’s syndrome and type 2 cryoglobulinaemia, in infection, and in healthy individuals. ACPAs, also called anti-cyclic citrullinated peptide (anti-CCP) antibodies, are reactive to the amino acid citrulline and are also present in the sera of patients with RA.^2^ The ACPA test is a laboratory-based enzyme-linked immunosorbent assay (ELISA). Point-of-care testing devices for both RF and ACPA are currently being developed.

## PATIENT GROUP AND USE

- Adult patients in primary care with suspected RA.

## IMPORTANCE

RA is a destructive inflammatory joint disease with an estimated UK prevalence of 1.2% in females and 0.4% in males.^3^ An individual GP is likely to see one new case of RA per year.^4^ Prompt presentation, recognition of symptoms and signs, and accurate interpretation of tests are likely to lead to better outcomes. NICE guidance on the management of RA (CG79) advises that the diagnosis should be suspected in patients presenting with synovitis of unknown cause; specifically symmetrical joints involvement of the hands and feet. In addition, pain, swelling, and stiffness (particularly in the morning), tender warm joints, a family history of RA, nodules and systemic features of malaise, fever, and weight loss should be considered as reasons to refer. NICE guidance explicitly advises against delaying urgent referral of ‘*any person with suspected persistent synovitis of undetermined cause whose blood tests show a normal acute-phase response or negative RF.*’

Many GPs undertake investigations including RF to distinguish patients with early RA from a larger number of patients with non-inflammatory joint pain. However, most studies on the diagnostic utility of RF are based in secondary care where the pre-test probability of RA is higher. In contrast, few studies have investigated the diagnostic utility of RF in primary care.^5^ Furthermore, studies suggest that RF results influence referral decisions and that GPs may use a negative RF result to exclude RA, despite the presence of appropriate symptoms.^5^ It is unclear whether ACPA instead of RF testing would lead to a higher diagnostic yield in primary care.

## PREVIOUS RESEARCH

### Accuracy compared to existing technology

Approximately 60–70% patients with RA have a positive RF, which is predictive of disease severity, but not so useful for diagnosis.^6^ Only 11–20% of people with musculoskeletal symptoms and a positive RF actually have RA.^6^

ACPA has similar sensitivity to RF but better specificity.^1^ A meta-analysis of 50 studies of RF and 37 of ACPA from both primary and secondary care populations reported pooled sensitivity for RF of 69% and specificity 85% (+LR 4.9; −LR 0.38).^7^ Pooled sensitivity of ACPA was estimated to be 67% and specificity 95% (+LR 12.5; −LR 0.36). Studies were based in hospital arthritis clinics and there are no large studies of RF or ACPA diagnostic utility based in primary care.

### Impact compared to existing technology

There is currently no evidence to support the use of RF or ACPA as diagnostic tests for RA in primary care. ACPA and RF are not useful in patients with a low pre-test probability of RA (<10%). In patients with a moderate pre-test probability (25–50%) the effect of a positive ACPA test is better than a positive RF. In patients with a high pre-test probability of RA, either test will perform well. Since both tests have poor sensitivities, negative results should not deter the clinician from a diagnosis of RA.^8^

One study investigated the outcome of a positive RF or ACPA in patients before the onset of RA symptoms.^9^ Out of 79 RA patients, 39 (49%) had RF and/or ACPA on at least one occasion before symptom onset. Analysis of the RF status in known RA cases showed a positive predictive value (PPV) of developing RA, 0–5 years before the onset of symptoms, of 88%. In contrast, the PPV was 97% with an initial positive ACPA result. But in healthy individuals, a positive RF test resulted in a 1.5% risk of developing RA in the subsequent 5 years, whereas a positive ACPA test had a 5.3% risk of developing RA.

A recent meta-analysis of three cohort studies where patients with early RA were tested for both RF and ACPA,^10^ showed that the positive likelihood ratio increased from 22.0 (95% confidence interval [CI] = 9.9 to 49.1) for ACPA alone to 27.1 (95% CI = 10.1 to 72.7) when both ACPA and RF results were positive. However, because of these large CIs, the authors were unable to conclude if adding ACPA testing to RF would significantly aid diagnosis. In addition, they also showed that there was no significant improvement to both sensitivity and specificity.

### Cost-effectiveness and economic impact

The cost-effectiveness of RF and ACPA should consider the impact of false-negative and false-positive results. A false-negative result may delay diagnosis and treatment resulting in additional subsequent costs to patients and the NHS. Similarly, unnecessary referrals to secondary care resulting from a false-positive result incur additional healthcare costs and also creates needless burden to individuals. There is little economic evidence about diagnostic procedures such as RF and ACPA in primary care. A recent study examined the cost-effectiveness of ACPA when compared to the American College of Rheumatology Criteria for the diagnosis of RA.^11^ The researchers reported a baseline cost per QALY gained estimate of €930 (£857) (2008 prices) indicating that ACPA is cost-effective using current thresholds of willingness to pay. The cost-effectiveness of RF versus ACPA or any other alternative has not been formally evaluated in the literature.

#### Relevant guidelines

NICE clinical guidance: *Rheumatoid arthritis: The management of rheumatoid arthritis in adults* (http://www.nice.org.uk/nicemedia/pdf/CG79NICEGuideline.pdf)

An American College of Rheumatology/European League Against Rheumatism Collaborative Initiative: *2010 Rheumatoid Arthritis Classification Criteria* (http://www.rheumatology.org/practice/clinical/classification/ra/2010_revised_criteria_classification_ra.pdf)

#### What this technology adds

Despite widespread use, the role of RF in diagnosing RA in primary care remains unclear. Newer tests, such as ACPA, are emerging with higher specificity and positive predictive values, but similar sensitivity. However, the value of these tests is in predicting a poorer prognostic group of secondary care patients with arthritis. Currently GPs should base diagnostic and referral decisions on clinical features; number and site of involved joints and elevated acute phase response, rather than serological tests. A positive RF or ACPA has value in supporting these decisions, but a negative test does not rule out disease.

#### Methodology

Standardised methodology was applied in writing this report, using prioritisation criteria and a comprehensive, standardised search strategy, and critical appraisal. Full details of these are available from www.madox.org.

#### Funding

This article presents independent research funded by the National Institute for Health Research (NIHR) under its Programme Grants for Applied Research funding scheme (RP-PG-0407-10347). The views expressed in this article are those of the author(s) and not necessarily those of the NHS, the NIHR or the Department of Health. Kamal R Mahtani is a NIHR academic lecturer.
